# Improvement in Pain, Quality of Life, and Urinary Dysfunction following Correction of Lumbar Lordosis and Reduction in Lumbar Spondylolistheses Using Chiropractic BioPhysics^®^ Structural Spinal Rehabilitation: A Case Series with >1-Year Long-Term Follow-Up Exams

**DOI:** 10.3390/jcm13072024

**Published:** 2024-03-30

**Authors:** Curtis A. Fedorchuk, Cole G. Fedorchuk, Douglas F. Lightstone

**Affiliations:** Institute of Spinal Health and Performance, Cumming, GA 30041, USA; cfedorchuk@comcast.net (C.A.F.); clcafedorchuk@comcast.net (C.G.F.)

**Keywords:** Chiropractic BioPhysics, spondylolisthesis, Mirror Image, lumbar spine, spinal rehabilitation, spinal traction, sagittal spinal alignment

## Abstract

**(1) Background:** Lumbar spondylolisthesis affects ~20% of the US population and causes spine-related pain and disability. **(2) Methods:** This series reports on three patients (two females and one male) aged 68–71 years showing improvements in back pain, quality of life (QOL), and urinary dysfunction following correction of lumbar spondylolistheses using CBP^®^ spinal rehabilitation. Pre-treatment radiographs showed lumbar hyperlordosis (−49.6°, ideal is −40°) and anterolisthesis (14.5 mm, ideal is 0 mm). Pre-treatment patient-reported outcome measures (PROMs) included a numeric rating scale (NRS) for back pain (7.3/10, ideal is 0), urinary urgency (8/10, ideal is 0), and SF-36 physical (PCS) and mental component score (MCS) (29.8 and 46.6, ideal is 46.8 and 52.8). Patients underwent 2–3 CBP^®^ sessions per week to correct lumbar hyperlordosis and lumbar anterolistheses. **(3) Results:** Post-treatment radiographs showed improvements in lumbar curvature (−42.8°) and anterolisthesis (4.2 mm). Post-treatment PROMs showed improvements in NRS for back pain (1/10), urinary urgency (2.3/10), and SF-36 PCS and MCS (50.2 and 57.7). Long-term follow-up radiographs and PROMs showed maintained improvements. **(4) Conclusions:** This series documents the first-recorded long-term corrections of lumbar spondylolisthesis and concomitant improvements in back pain, urinary urgency, and QOL using CBP^®^. This series provides evidence for CBP^®^ as a non-surgical approach to lumbar spinal rehabilitation and the possible impacts of spinal alignment on pain, urinary dysfunction, and QOL.

## 1. Introduction

Chronic spinal pain accounts for the greatest global burden of disease (GBD), and years lived with disability (YLD) is an indicator for dysfunction, disability, and decreased quality of life (QOL) and is an increasing problem worldwide [1]. In addition, neuromusculoskeletal conditions may further manifest as an alteration in autonomic nervous system function. This somato-autonomic/visceral reflex/effect may lead to visceral function changes in dependent organs or organ systems [2]. The effective treatment and diagnosis of spine-related neuromusculoskeletal conditions and pathologies, including lumbar spondylolisthesis, require a biomechanical assessment of the spine [3].

Spondylolisthesis is characterized by the translational displacement of one vertebra in relation to the vertebra beneath it [4]. It is a vertebral subluxation characterized by a kinesiologic dysfunction and neurological involvement [5]. Degenerative spondylolisthesis is prevalent in 6–31% of the United States population [6,7,8], with an elevated prevalence from 50 to 90 years of age and where the female to male ratio is 5 to 1 [6,9]. Increased age, estrogen production, facet sagittalization, lumbar hyperlordosis, increased body mass index (BMI), and past pregnancies have been suggested as predisposing factors for females [7,9,10].

There are surgical and non-surgical treatments for spondylolisthesis that focus on stabilization of the spine [11]. Various surgical procedures have been used to stabilize and reduce lumbar spondylolisthesis, including decompression with and without fusion, instrumented and non-instrumented fusion, open and minimally invasive surgery (MIS), and interbody (e.g., (anterior lumbar interbody fusion (ALIF), lateral lumbar interbody fusion (LLIF), posterior lumbar interbody fusion (PLIF), and transforaminal lumbar interbody fusion (ALIF)) and no interbody surgery [12]. These procedures have been studied and compared for efficacy clinical success [12]. Research suggests that surgical interventions for lumbar spondylolisthesis provide greater clinical success compared with non-surgical approaches [11]. However, these studies compared surgical measures that stabilized and corrected the lumbar spondylolistheses to non-surgical interventions that have not been shown to stabilize or correct lumbar spondylolisthesis. The outcome measures included QOL assessments, pain scales, disability indices, and satisfaction questionnaires.

Non-surgical methods include active physical therapy, education or counseling for exercising, nonsteroidal anti-inflammatory drugs (NSAIDs), homeopathic remedies, soft tissue massage, trigger point therapy, spinal mobilization techniques to restricted areas, cryotherapy, and chiropractic methods [11,13,14,15,16]. Although non-operative treatment for spondylolisthesis remains scarce in the literature, patients receiving non-surgical treatment have shown significant improvements [11]. However, until recently, these non-surgical methods have not been shown to correct lumbar spondylolisthesis. In this sense, a non-surgical spinal rehabilitation protocol (Chiropractic BioPhysics^®^ (CBP^®^)) has resulted in successful corrections of spondylolisthesis [17,18,19]. In 2017, Fedorchuk et al. provided the first documented evidence of a non-surgical, structural spinal rehabilitation, reducing an L4 anterolisthesis from 13.3 mm to 2.4 mm in a 69-year-old female [18]. The patient was suffering moderate lower back pain and severe leg cramping, being resealed after completing 60 sessions of CBP^®^ Mirror Image^®^ (MI) spinal exercises, adjustments, and traction over 45 weeks. A similar protocol based on CBP^®^, including MI lumbar spine drop-table adjustments, corrective exercises, and a unique lumbopelvic spinal traction, was performed 50 times over 7 months on a 57-year-old man [19]. A radiograph showed full reduction for both the L3 retrolisthesis and L4 anterolisthesis after 3 and 7 months [19].

Conservative, non-surgical, rehabilitation methods for the correction of spondylolistheses are underrepresented in the literature. This study provides data from three new cases documenting improvements in pain, QOL, and urinary dysfunction following CBP^®^ structural spinal rehabilitation of lumbar spondylolistheses, as previously reported [18,19]. However, this study also includes a long-term follow-up of greater than 1 year following treatment, which shows stabilization of the corrected spondylolistheses. This study and others like it are necessary to set the stage for more advanced studies and higher levels of evidence to compare to surgical methods that achieve the same biomechanical outcomes.

## 2. Materials and Methods

This case series does not involve any experimentation on human or animal subjects. Patients were treated according to standards of practice and practice guidelines. The patients were provided with and completed informed consent for publication of their health information and case results. All procedures performed in this study were in accordance with the ethical standards of the 1964 Helsinki Declaration and its later amendments or comparable ethical standards.

### 2.1. Patient Inclusion and Exclusion Criteria

This case series reports on three patients who reported to a spinal rehabilitation facility for low back pain. For all patients, the following points were consistent at their initial visit:

- Health histories revealed disorder of the urinary system (International Classification of Diseases, Tenth Revision (ICD-10) N39.9) and urgency of urination (ICD R39.15).
- Physical examination revealed low back pain (ICD-10 M54.50) and lumbar radiculopathy (ICD-10 M54.16).
- Patient-reported outcomes (PROs) and measures (PROMs) using short-form 36-question health-related quality of life (HRQOL) questionnaire (SF-36) revealed decreased quality of life, and numeric rating scale (NRS) revealed moderate-to-severe low back pain and severe urinary urgency.
- Sagittal radiographic exams revealed lumbar hyperlordosis (ICD-10 M40.56) and spondylolistheses of the lumbar spine (ICD-10 M43.16).

In this case series, patients were excluded if they were not compliant with CBP^®^ spinal rehabilitation treatment recommendations (e.g., treatment consistency, duration, etc.) or if the patients did not complete a post-treatment or long-term follow-up exam.

### 2.2. Patient-Reported Outcomes and Measures

High-quality clinical care requires patients to provide information regarding their symptoms and condition. These patient-reported outcomes (PROs) may provide qualitative (e.g., mild, moderate, severe, etc.) or quantitative (e.g., on a scale of 0–10, 0–100, etc.) measures. Patient-reported outcome measures (PROMs) are the tools or instruments used to measure PROs. These tools are often patient-completed and measure functional status, HRQOL, and symptom and symptom burden for general health or specific health conditions [20].

#### 2.2.1. Numeric Rating Scale

An NRS is a PROM that allows patients to quantify the severity of a given condition. The NRS provides a scale from 0 to 10, where 0 represents no symptom and 10 represents the maximum severity of a symptom. For pain, the NRS is a valid and reliable PROM [21] that patients report as comprehensive and simple to understand and complete [21]. On the 0-to-10 scale, pain quality per quantity is none at 0, mild at 1 to 3, moderate at 4 to 7, and severe at 8 to 10 [22]. For urinary urgency, the NRS has been used in research [23] and has content validity as it is consistent with the patient perception of intensity of urgency scale (PPIUS) [24]. On the 0-to-10 scale, urinary urgency quality per quantity is none at 0, mild at 1 to 3, moderate at 4 to 6, severe at 7 to 9, and incontinence at 10 [22].

The patients in this case series were asked to report on the severity of their back pain and urinary urgency using an NRS for each. The minimally clinically important difference (MCID) needs to be −2 points on the NRS to represent a significant improvement in pain [25].

#### 2.2.2. Short-Form 36-Question Health-Related Quality-of-Life Questionnaire

The SF-36 questionnaire comes from the Medical Outcomes Study [26] and is a valid, reliable PROM that assesses HRQOL factors based on healthcare outcomes and is the most utilized HRQOL assessment in research [27]. The SF-36 has been shown to be valid for physical dysfunction in seniors [28] and community-dwelling older adults [29]. SF-36 validity is supported by correlations between SF-36 scales and measures of disease symptoms, activity, and functioning [30].

The SF-36 provides scaled scores for nine domains on a scale of 0 to 100, where 0 is the lowest score and 100 is the highest score. These domains are as follows: physical functioning (PF), role limitations due to physical health (RP), role limitations due to emotional problems (RE), vitality (energy/fatigue) (VT), mental health (MH), social functioning (SF), bodily pain (BP), general health (GH), and change in health (ΔH). A change of 20 points or more represents a significant change in domain scores. From the domains, summative scores can be calculated, providing a physical component score (PCS) and a mental component score (MCS) [31]. A change of 6 points or more represents a significant change in the summative component scores [32].

### 2.3. Radiographic Analysis

Spinal radiographs were taken with the patient in a normal, neutral, upright, weight-bearing position, allowing us to detect alignment abnormalities (i.e., rotations and translations of the head, rib cage, and pelvis from a normal position in a 3-dimensional coordinate system) [33].

The radiographs of the patients were examined using the Harrison posterior tangent method (HPTM) [34]. Lateral lumbar (LL) radiographs are used to measure regional and intervertebral lumbar angles and translations. Lumbar angles are determined by drawing a tangential line to the posterior aspect of each vertebral body from T12 to S1. The absolute rotation angle (ARA) is assessed for spinal regions (e.g., ARA L1-L5). Intervertebral AP translations are determined by measuring the perpendicular distance from the posterior tangent line of the inferior vertebra to the posterior, inferior aspect of the superior vertebra. All measurements and lines are compared to established, evidence-based, and normative values.

Spinal radiographic analysis involving the Cartesian coordinate system to assess translations and rotations of the cervical, thoracic, and lumbopelvic spine in and around the x, y, and z axes was performed using PostureRay^®^ radiographic electronic medical records (EMR) software version PostureRay v27a18 (PostureCo, Inc., Trinity, FL, USA) [35,36].

### 2.4. Patients’ Presentations

#### 2.4.1. Patient 1

**Figure 1:**
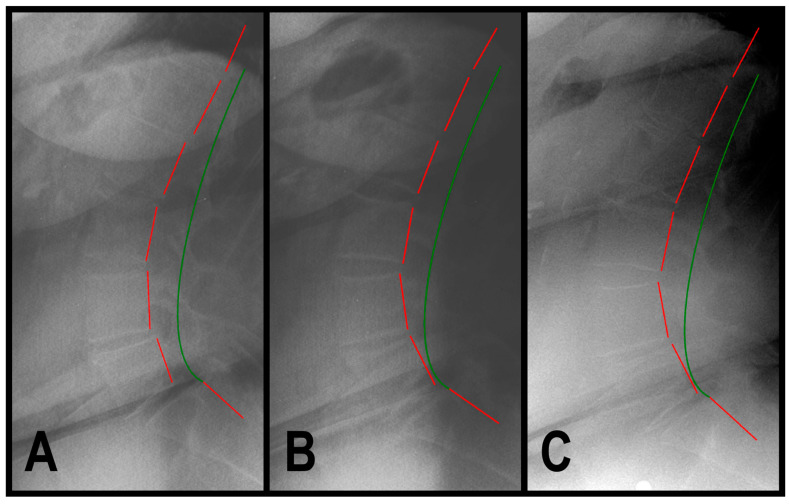
(**A**–**C**) Pre-treatment, post-treatment, and 4-year, long-term follow-up radiography neutral lateral lumbar radiographs of a 71-year-old female initially reporting 7/10 low back pain with pain radiating into her left leg. The green line represents a normal, ideal sagittal cervical alignment and the red line represents the actual posterior tangent lines of the T12-S1 vertebrae. (**A**) Pre-CBP^®^ treatment neutral lateral lumbar radiograph in 9/2019 with an ARA L1-L5 measuring −47.8° (40° is ideal) and an L5 anterolisthesis Tz L5-S1 measuring 15.8 mm (ideal is 0 mm, lumbar spondylolisthesis is >4.5 mm); (**B**) post-CBP^®^ treatment neutral lateral lumbar radiograph in 12/2019 with improvement in ARA L1-5 measuring −41.1° and Tz L5-S1 measuring 4.2 mm; and (**C**) 4-year follow-up post-CBP^®^ treatment neutral lateral lumbar radiograph in 1/2024 with maintained improvement in ARA L1-L5 measuring −43.4° and Tz L5-S1 measuring 4.3 mm.

A 71-year-old female (170.2 cm height, 86.2 kg) presented to a spinal rehabilitation facility in 9/2019, reporting constant, aching, moderate low back pain that she rated 7/10 on a pain NRS of 0–10, where 0 is no pain and 10 is maximum pain, and with pain radiating into her left leg. SF-36 assessment revealed decreased QOL with a summative physical component score (PCS) of 32.6 (normal is 46.8) and a mental component score (MCS) of 53.8 (normal is 52.8) (Table 1). A sagittal lumbar radiographic exam revealed hyperlordosis of the lumbar spine curvature from L1 to L5 (ARA L1-L5) measuring −47.8° (ideal is −40°) and anterolisthesis of L5 on S1 (Tz L5-S1) measuring 15.8 mm (ideal is 0 mm) (Table 1, Figure 1A). The health history revealed normal urination frequency of 6 times in a 24 h period; however, there was severe urinary urgency the patient rated 8/10 on an NRS of 0 to 10, where 0 is complete control of bladder/urination and 10 is no control of bladder/urination (Table 1).

**Figure 2:**
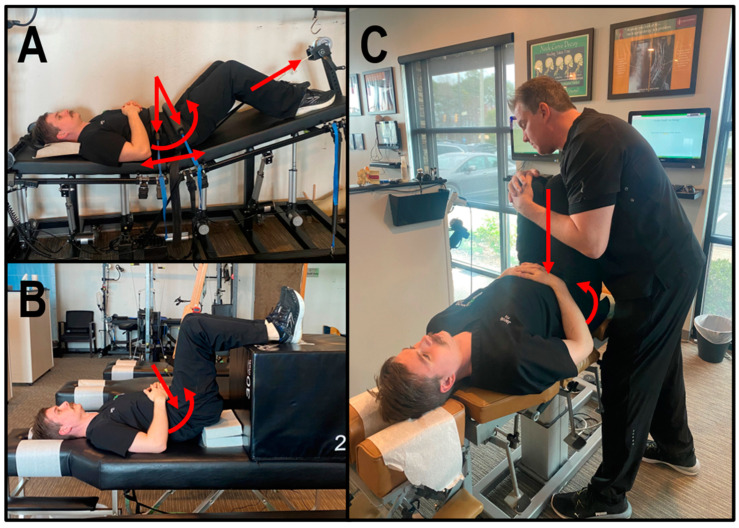
(**A**–**C**) Chiropractic BioPhysics^®^ structural spinal rehabilitation Mirror Image^®^ spinal traction, therapeutic exercises, and chiropractic adjustment. The red arrow represents prescribed movements/forces for MI structural spinal rehabilitation treatment using CBP^®^ protocols. All therapies involved reducing lumbar lordosis by leveraging the pelvis into flexion (-RxP) to induce lumbar flexion reducing the L5 anterolisthesis (**A**) MI spinal traction including lumbar decompression; (**B**) MI spinal exercises; and (**C**) MI chiropractic adjustments.

Patient 1 underwent 36 treatment sessions over 12 weeks of CBP^®^ structural spinal rehabilitation, including MI spinal therapeutic exercises, mechanical traction, and chiropractic adjustments. All therapies involved reducing lumbar lordosis by leveraging the pelvis into flexion (-RxP) to induce lumbar flexion, reducing the L5 anterolisthesis (Figure 2A–C).

#### 2.4.2. Patient 2

**Figure 3:**
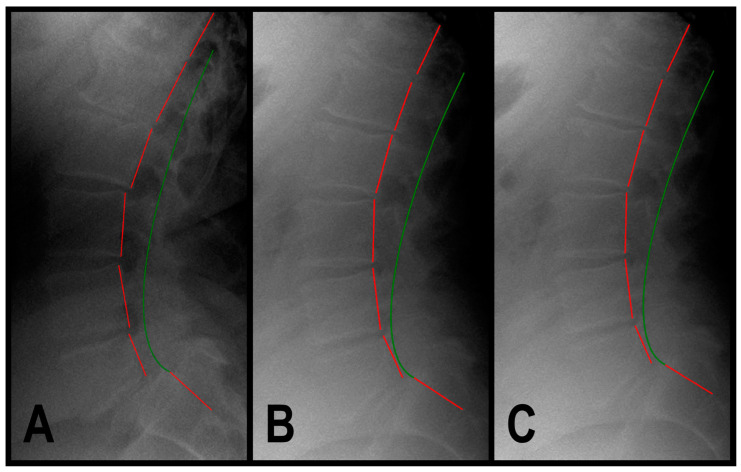
(**A**–**C**) Pre-treatment, post-treatment, and 4-year, long-term follow-up radiography neutral lateral lumbar radiographs of a 68-year-old male initially reporting 8/10 low back pain with pain radiating into his right buttock and leg. The green line represents a normal, ideal sagittal cervical alignment and the red line represents the actual posterior tangent lines of the T12-S1 vertebrae. (**A**) Pre-CBP^®^ treatment neutral lateral lumbar radiograph in 2/2022 with an ARA L1-L5 measuring −49.9° (40° is ideal) and an L5 anterolisthesis Tz L5-S1 measuring 13.8 mm (ideal is 0 mm, lumbar spondylolisthesis is >4.5 mm); (**B**) post-CBP^®^ treatment neutral lateral lumbar radiograph in 5/2022 with improvement in ARA L1-L5 measuring −42.2° and Tz L5-S1 measuring 4.4 mm; and (**C**) 1.25-year follow-up post-CBP^®^ treatment neutral lateral lumbar radiograph in 8/2023 with maintained improvement in ARA L1-L5 measuring −43.6° and Tz L5-S1 measuring 4.4 mm.

A 68-year-old male (177.8 cm height, 117.9 kg) presented to a spinal rehabilitation facility in 2/2022, reporting constant, sharp, and dull ache, with severe low back pain that he rated 8/10 on a pain NRS with pain radiating into his right buttock and leg. SF-36 assessment revealed decreased QOL with a PCS of 21.8 and an MCS of 42.2 (Table 2). Sagittal lumbar radiographic exam revealed ARA L1-L5 measuring −49.9° and Tz L5-S1 measuring 13.8 mm (Table 2, Figure 3A). Health history revealed increased urination frequency of 12 times in a 24 h period (normal is 6–7 times in a 24 h period), and there was severe urinary urgency the patient rated 9/10 on an NRS (Table 2).

Patient 2 underwent 36 treatment sessions over 12 weeks of CBP^®^ structural spinal rehabilitation, including MI spinal therapeutic exercises, mechanical traction, and chiropractic adjustments. All therapies involved reducing lumbar lordosis by leveraging the pelvis into flexion to induce lumbar flexion, reducing the L5 anterolisthesis (Figure 2A–C).

#### 2.4.3. Patient 3

**Figure 4:**
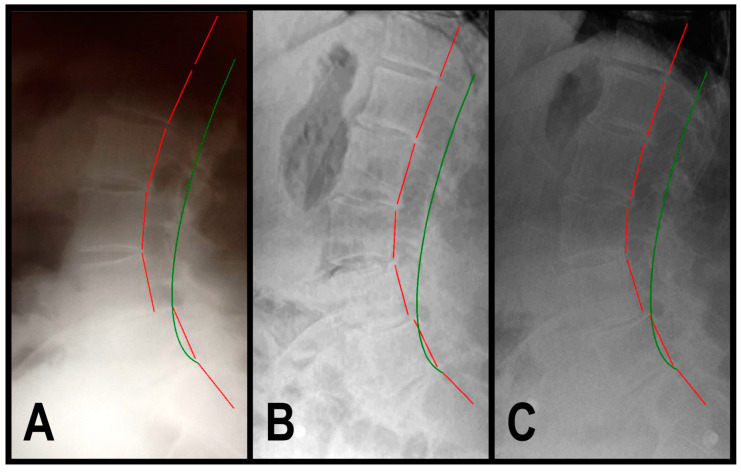
(**A**–**C**) Pre-treatment, post-treatment, and 4-year, long-term follow-up radiography neutral lateral lumbar radiographs of a 69-year-old female initially reporting 7/10 low back pain with pain radiating into her right leg. The green line represents a normal, ideal sagittal cervical alignment and the red line represents the actual posterior tangent lines of the T12-S1 vertebrae. (**A**) Pre-CBP^®^ treatment neutral lateral lumbar radiograph in 3/2015 with an ARA L1-L5 measuring −51.2° (40° is ideal) and an L4 anterolisthesis Tz L4-L5 measuring 13.5 mm (ideal is 0 mm, lumbar spondylolisthesis is >4.5 mm); (**B**) post-CBP^®^ treatment neutral lateral lumbar radiograph in 1/2016 with improvement in ARA L1-L5 measuring −45.2° and Tz L4-L5 measuring 2.6 mm; and (**C**) 3.75-year follow-up post-CBP^®^ treatment neutral lateral lumbar radiograph in 8/2019 with maintained improvement in ARA L1-L5 measuring −45.4° and Tz L4-L5 measuring 4.2 mm.

A 69-year-old female (160.0 cm height, 59.8 kg) presented to a spinal rehabilitation facility in 3/2015, reporting constant, dull, throbbing, moderate low back pain that she rated 7/10 on a pain NRS with cramping pain radiating into her right leg. SF-36 assessment revealed decreased QOL, with a PCS of 35.0 and an MCS of 43.5 (Table 3). Sagittal lumbar radiographic exam revealed ARA L1-L5 measuring −51.2° and Tz L4–L5 measuring 13.5 mm (Table 3, Figure 4A). Health history revealed normal urination frequency of 6 times in a 24-h period; however, there was severe urinary urgency the patient rated 7/10 on an NRS (Table 3).

Patient 3 underwent 65 treatment sessions over 41 weeks of CBP^®^ structural spinal rehabilitation, including MI spinal therapeutic exercises, mechanical traction, and chiropractic adjustments. All therapies involved reducing lumbar lordosis by leveraging the pelvis into flexion to induce lumbar flexion, reducing the L4 anterolisthesis (Figure 2A–C).

#### 2.4.4. Summary of Patients’ Presentations

Pre-treatment radiographic exams’ findings included lumbar hyperlordosis, with a mean lumbar curvature measuring −49.6° (ideal is −40°) and mean translation of a lumbar anterolisthesis measuring 14.5 mm (ideal is 0). PROMs included mean back pain numeric rating scale (NRS) of 7.3/10 (ideal is 0) indicating moderate-to-severe low back pain, mean urinary urgency NRS of 8/10 (ideal is 0) indicating severe urinary urgency, and mean SF-36 PCS of 29.8 (ideal is 46.8) and MCS of 46.67 (ideal is 52.8). Patients underwent 2 to 3 treatment sessions per week of CBP^®^ structural spinal rehabilitation, including Mirror Image^®^ spinal exercises, traction, and chiropractic adjustments. All therapies involved reducing lumbar lordosis by leveraging the pelvis into flexion to induce lumbar flexion, reducing the lumbar anterolistheses towards normal spinal measures.

### 2.5. Chiropractic BioPhysics^®^ Structural Spinal Rehabilitation

Patients underwent multiple treatment sessions per week of CBP^®^ structural spinal rehabilitation, including MI spinal therapeutic exercises, mechanical traction, and chiropractic adjustments. All therapies involved reducing lumbar lordosis by leveraging the pelvis into flexion to induce lumbar flexion, reducing the L4 anterolisthesis towards normal spinal measures (Figure 2A–C) [37,38,39,40,41].

#### 2.5.1. Mirror Image^®^ Spinal Traction

Based on successful experiences and the existing literature [18,33,42], lumbar traction was administered to three distinct patients using the Robo-Trac Decompression and Traction Unit (Advanced Spinal Rehab, Middletown, NY, USA). Each patient, positioned supine, underwent targeted pelvis flexion with elevation of the legs and knees flexed, and an anterior-to-posterior (A-P) force at the anterior superior iliac spines (ASIS) of the pelvis and the lower abdomen to allow for leveraging of the pelvis into flexion to induce lumbar flexion, reducing the L4 anterolisthesis (Figure 2A). The traction sessions commenced with each patient enduring traction to their tolerance for 2 to 4 min, progressively increasing by 2 min per visit until achieving 15 to 20 min.

#### 2.5.2. Mirror Image^®^ Spinal Exercises

MI exercises involve strengthening weakened muscles and elongating tight muscles that have adjusted to poor posture to correct and sustain improvements in spinal alignment and address postural abnormalities [33]. Patients were instructed to lay supine with their legs raised in a 90-degree flexed hip and knee position. Blocks are placed under the sacrum, creating a fulcrum at S2/3 to allow for leveraging of the pelvis into flexion to induce lumbar flexion, reducing the L4 anterolisthesis (Figure 2B). Patients were guided to contract for 3 to 5 s and then relax for 3 s for 10 min per session.

#### 2.5.3. Mirror Image^®^ Adjustments

MI adjustments were administered to each patient using an OMNI elevation table equipped with sectional drop mechanisms. These drop-table adjustments aim to activate mechanoreceptors and proprioceptors [42], crucial for conveying body position information to the brain and maintaining spatial awareness [43]. By stimulating these sensory receptors during adjustments in the MI position, the goal is to retrain the central nervous system (CNS) to conform to the normal spinal posture [42].

Each patient received A-P adjustments while lying supine with pelvis and knees flexed and a fulcrum at S2/3 to allow for leveraging of the pelvis into flexion to induce lumbar flexion, reducing the L4 anterolisthesis (Figure 2C). Adjustments involved applying downward force through the legs using the lumbar drop mechanism, facilitating the correction process.

## 3. Results

### 3.1. Post-Treatment and Long-Term Follow-Up Exam Results

Post-treatment exams were performed at least 24 h after the previous treatment session to eliminate any short-term biomechanical effects of the rehabilitation from affecting the results. Long-term follow-up exams were performed after a minimum of 1.25 years among the three patients, during which no structural spinal rehabilitation treatments were administered. Post-treatment and long-term follow-up SF-36, pain and urinary urgency NRSs, and radiographic exams were performed.

#### 3.1.1. Patient 1

In 12/2019, a post-treatment exam revealed improvement in back pain NRS to 1/10 (mild) and SF-36 PCS to 51.7 and MCS to 57.5 (Table 1). The patient described her pain as occasional and minimal (1/10). The sagittal lumbar radiographic exam revealed a correction of ARA L1-L5 measuring −41.1° and Tz L5-S1 measuring 4.2 mm (Table 1, Figure 1B). The patient stated that her urinary urgency was mild, which she rated 2/10 (mild) (Table 1).

In 1/2024, a 4-year long-term follow-up exam revealed a maintained improvement in back pain and QOL (Table 1). The sagittal lumbar radiographic exam revealed a maintained correction of ARA L1-L5 measuring −43.4° and Tz L5-S1 measuring 4.3 mm (Table 1, Figure 1C). The patient reported maintained improvements in her urinary urgency (Table 1).

#### 3.1.2. Patient 2

In 5/2022, a post-treatment exam revealed improvements in back pain NRS to 1/10 (mild) and SF-36 PCS to 44.1 and MCS to 62.7 (Table 2). The patient described his pain as nearly resolved and minimal (1/10). The sagittal lumbar radiographic exam revealed a correction of ARA L1-L5 measuring −42.2° and Tz L5-S1 measuring 4.4 mm (Table 2, Figure 3B). The patient reported improvements in urinary frequency to six times in 24 h and that his urinary urgency was very tolerable, rated as 3/10 (mild) (Table 2).

In 8/2023, a 1.25-year long-term follow-up exam revealed a maintained improvement in back pain and QOL (Table 2). The sagittal lumbar radiographic exam revealed a maintained correction of ARA L1-L5 measuring −43.6° and Tz L5-S1 measuring 4.4 mm (Table 2, Figure 3C). The patient reported maintained improvement in his urinary frequency and urgency (Table 2).

#### 3.1.3. Patient 3

In 1/2016, a post-treatment exam revealed improvement in back pain NRS to 1/10 and SF-36 PCS to 54.8 and MCS to 52.7 (Table 3). The patient described her pain as nearly resolved and minimal (1/10). The sagittal lumbar radiographic exam revealed a correction of ARA L1-L5 measuring −45.2° and Tz L4-L5 measuring 2.6 mm (Table 3, Figure 4B). The patient stated that her urinary urgency was mild, rated as 2/10 (mild) (Table 3).

In 8/2019, a 3.75-year long-term follow-up exam revealed maintained improvement in back pain and QOL (Table 3). The sagittal lumbar radiographic exam revealed a maintained correction of ARA L1-L5 measuring −45.4° and Tz L4-L5 measuring 4.2 mm (Table 3, Figure 4C). Of note, Patient 3 stated that she sustained a motor vehicle collision in 2017. The sagittal lumbar radiograph showed maintained stability, despite the motor vehicle crash trauma. The patient reported maintained improvement in her urinary urgency (Table 3).

#### 3.1.4. Summary of Patients’ Post-Treatment and Long-Term Follow-Up Exam Results

The post-treatment radiographic exams’ findings included improvements in mean lumbar curvature from −49.6° to −42.8° and mean translation of a lumbar anterolisthesis from 14.5 mm to 4.2 mm. Post-treatment PROMs showed improvements in mean back pain NRS from 7.3/10 (moderate-to-severe) to 1/10 (mild), mean urinary urgency NRS from 8/10 (severe) to 2.3/10 (mild), and mean SF-36 PCS from 29.8 to 50.2 and MCS from 46.7 to 57.7. Long-term follow-up radiographic exams revealed a maintained correction of lumbar curvature measuring −44.1° and mean translation of a lumbar anterolisthesis measuring 4.3 mm. Long-term follow-up PROMs maintained improvements in mean back pain NRS at 2/10 (mild), mean urinary urgency NRS at 2.33/10 (mild), and with mean SF-36 PCS at 48.9 and MCS at 57.2.

## 4. Discussion

This case documents the first recorded long-term successful treatment of three senior patients (two females and one male) aged 68 to 71 years showing improvements in back pain, QOL, and urinary dysfunction following the correction of lumbar lordosis and reduction in lumbar spondylolistheses using CBP^®^ structural spinal rehabilitation. This case provides evidence showing the impact of spinal alignment on pain (somato-somatic reflex/effect), urinary dysfunction (somato-autonomic/visceral reflex/effect), and HRQOL.

Although non-operative treatment for degenerative spondylolisthesis remains widely underrepresented in the literature, these approaches encompass a range of methods, including active physical therapy, exercise education and counseling, NSAIDs, homeopathic remedies, soft tissue massage, trigger point therapy, spinal mobilization techniques targeting restricted areas, cryotherapy, and chiropractic interventions, among others. Studies show a correlation between increased lumbar lordosis and lumbar spondylolistheses prevalence [44,45,46]. Previous works demonstrate the benefits of CBP^®^ structural spinal rehabilitation [17,18,19,42]. Our long-term follow-up results align with previous studies, reinforcing the consistent average improvements observed in patients with image-confirmed degenerative spondylolisthesis [11,47,48]. Our findings also contribute to the growing body of evidence indicating that there may not be a significant advantage to surgery compared to non-surgical care in this patient population [11].

CBP^®^ structural spinal rehabilitation treats the abnormal spinal alignment associated with degenerative spondylolisthesis through the application of mechanical traction, therapeutic exercises, and chiropractic adjustments using combinations of over-corrective MI biomechanical forces. The activation of mechanoreceptors and proprioceptors responsible for transmitting the body’s position to the brain enable it to comprehend the body’s spatial orientation [43]. Stimulating these sensory receptors during MI therapies reconditions the nervous system to align with the healthy spinal alignment and posture [1], and MI traction induces plastic viscoelastic deformation of the spinal ligaments, allowing for sustained structural corrections [33]. This process results in a lasting restorative change, as prolonged deformation forces act on the soft tissues accustomed to abnormal spinal posture, restoring a healthy spinal alignment [49].

Correcting spinal alignment and posture is the primary goal of CBP^®^ to help reduce abnormal spinal, paraspinal, and extraspinal tissue loads to improve pain and function. CBP^®^ has multiple studies in the scientific literature demonstrating short-term and long-term improvements in subjective patient-reported outcomes and QOL measures following the correction of spinal alignment and posture. CBP^®^ treatment across many various spinal and extraspinal conditions has shown objective and repeatable improvements in sagittal and coronal spinal alignment and postural balance using repeatable, reliable, and valid alignment-measuring technology, showing improved patient-reported outcomes [50].

This case series is limited in being able to draw conclusions about correlation, causation, or applying the patients’ findings to broader spectra of varying demographics. While there are many similarities among the patients in this case series, it is important to interpret results within the clinical context, considering the unique characteristics of each patient’s condition and the longitudinal nature of the follow-up assessments. As is true for case series, this study is further limited by sampling bias. Larger studies and clinical trials involving patients with lumbar spondylolisthesis (anterolisthesis), structural spinal rehabilitation, and control groups with long-term follow-ups are needed.

## 5. Conclusions

This case documents the long-term successful treatment of three senior patients showing improvements in back pain, QOL, and urinary dysfunction following the correction of lumbar lordosis and reductions in lumbar spondylolistheses using CBP^®^ structural spinal rehabilitation. This case provides evidence showing the impact of spinal alignment on pain, urinary dysfunction, and HRQOL.

This study adds to the growing body of research for healthcare providers and educators who can benefit from a better understanding of the impact of spinal health on human physiology and potential treatment options. Understanding spinal biomechanics may improve complicated cases such as the patients in this report. Improving subjective and objective outcomes contributes to lessening the GBD and YLD.

## Figures and Tables

**Table 1:** Pre-treatment, post-treatment, and 4-year, long-term follow-up health outcome assessments of a 71-year-old female initially reporting 7/10 low back pain with pain radiating into her left leg.

Assessment		Normal Value	Pre-Treatment Exam 9/2019	Post-Treatment Exam 12/2019	4-Year Follow-Up Exam 1/2024
Back Pain NRS		0	7	1	2
SF-36 HRQOL Scales	PF	72.0	45.0	85.0	80.0
	RP	81.0	25.0	100.0	100.0
	RE	81.0	66.7	100.0	100.0
	VT	61.0	40.0	60.0	55.0
	MH	81.0	76.0	92.0	96.0
	SF	83.0	87.5	100.0	100.0
	BP	75.0	45.0	90.0	87.5
	GH	72.0	67.5	80.0	80.0
	ΔH	84.0	25.0	75.0	75.0
	PCS	46.8	32.6	51.7	49.9
	MCS	52.8	53.8	57.5	58.7
ARA L1-L5 (°)		−40	−47.8	−41.1	−43.4
Tz L5-S1 (mm)		0	15.8	4.2	4.3
Urination Frequency (times/24 h)		0	6	6	6
Urinary Urgency NRS		0	8	2	2

Gray background color = normal values, PF = physical functioning, RP = role limitations due to physical health, RE = role limitations due to emotional problems, VT = vitality (energy/fatigue), MH = mental health, SF = social functioning, BP = bodily pain, GH = general health, ΔH = change in health, PCS = physical component score, MCS = mental component score, ° = degree(s), Tz = translation in the sagittal plane, mm = millimeter(s), h = hour(s).

**Table 2:** Pre-treatment, post-treatment, and 1.25-year, long-term follow-up health outcome assessments of a 68-year-old male initially reporting 8/10 low back pain with pain radiating into his right buttock and leg.

Assessment		Normal Value	Pre-Treatment Exam 2/2022	Post-Treatment Exam 5/2022	1.25-Year Follow-Up Exam 8/2023
Back Pain NRS		0	8	1	2
SF-36 HRQOL Scales	PF	72.0	0.0	55.0	55.0
	RP	81.0	0.0	100.0	100.0
	RE	81.0	0.0	100.0	100.0
	VT	61.0	15.0	80.0	75.0
	MH	81.0	80.0	100.0	80.0
	SF	83.0	25.0	75.0	100.0
	BP	75.0	22.5	70.0	62.5
	GH	72.0	67.5	90.0	90.0
	ΔH	84.0	25.0	100.0	75.0
	PCS	46.8	21.8	44.1	45.4
	MCS	52.8	42.2	62.7	60.1
ARA L1-L5 (°)		−40	−49.9	−42.2	−43.6
Tz L5-S1 (mm)		0	13.8	4.2	4.3
Urination Frequency (times/24 h)		0	12	6	6
Urinary Urgency NRS		0	9	3	3

Gray background color = normal values, PF = physical functioning, RP = role limitations due to physical health, RE = role limitations due to emotional problems, VT = vitality (energy/fatigue), MH = mental health, SF = social functioning, BP = bodily pain, GH = general health, ΔH = change in health, PCS = physical component score, MCS = mental component score, ° = degree(s), Tz = translation in the sagittal plane, mm = millimeter(s), h = hour(s).

**Table 3:** Pre-treatment, post-treatment, and 3.75-year, long-term follow-up health outcome assessments of a 69-year-old female initially reporting 7/10 low back pain with pain radiating into her right leg.

Assessment		Normal Value	Pre-Treatment Exam 3/2015	Post-Treatment Exam 1/2016	3.75-Year Follow-Up Exam 8/2019
Back Pain NRS		0	7	1	2
SF-36 HRQOL Scales	PF	72.0	50.0	95.0	85.0
	RP	81.0	25.0	80.0	75.0
	RE	81.0	66.7	90.0	100.0
	VT	61.0	40.0	70.0	70.0
	MH	81.0	52.0	76.0	72.0
	SF	83.0	60.0	100.0	87.5
	BP	75.0	50.0	90.0	87.5
	GH	72.0	50.0	85.0	80.0
	ΔH	84.0	25.0	75.0	75.0
	PCS	46.8	35.0	54.8	51.4
	MCS	52.8	43.5	52.7	52.8
ARA L1-L5 (°)		−40	−51.2	−45.2	−45.4
Tz L5-S1 (mm)		0	13.8	4.2	4.3
Urination Frequency (times/24 h)		0	6	6	6
Urinary Urgency NRS		0	7	2	2

Gray background color = normal values, PF = physical functioning, RP = role limitations due to physical health, RE = role limitations due to emotional problems, VT = vitality (energy/fatigue), MH = mental health, SF = social functioning, BP = bodily pain, GH = general health, ΔH = change in health, PCS = physical component score, MCS = mental component score, ° = degree(s), Tz = translation in the sagittal plane, mm = millimeter(s), h = hour(s).

## Data Availability

The original contributions presented in the study are included in the article material. Further inquiries can be directed to the corresponding author. Data shared are in accordance with consent provided by participants. The publication of such data does not compromise the anonymity of the participants or breach local data protection laws.
